# Molecular Pathology of Advanced NSCLC: Biomarkers and Therapeutic Decisions

**DOI:** 10.3390/cancers18020216

**Published:** 2026-01-09

**Authors:** Melanie Winter, Jan Jeroch, Maximilian Wetz, Marc-Alexander Rauschendorf, Peter J. Wild

**Affiliations:** 1 Dr. Senckenberg Institutes of Pathology and Human Genetics, University Hospital Frankfurt, Goethe University Frankfurt, Theodor-Stern-Kai 7, 60596 Frankfurt am Main, Germany; 2 University Hospital Frankfurt MVZ GmbH, Theodor-Stern-Kai 7, 60596 Frankfurt am Main, Germany; 3 Molecular Health, Kurfuersten-Anlange 21, 69115 Heidelberg, Germany; marc.rauschendorf@molecularhealth.com

**Keywords:** NSCLC, *EGFR*, *KRAS*, *ALK*, *ROS*, *PDL1*, personalized therapy

## Abstract

**Background:** Advances in molecular pathology have transformed NSCLC (Non-Small Cell Lung Cancer) diagnosis, prognosis, and treatment by enabling precise tumor characterization and targeted therapeutic strategies. We review key genomic alterations in NSCLC, including *EGFR* (epidermal growth factor receptor) mutations, *ALK* (anaplastic lymphoma kinase) and *ROS1* (ROS proto-oncogene 1) rearrangements, *BRAF* (B-Raf proto-oncogene serine/threonine kinase) mutations, *MET* (mesenchymal–epithelial transition factor) alterations, *KRAS* (Kirsten rat sarcoma) mutations, *HER2* (human epidermal growth factor receptor 2) alterations and emerging *NTRK* (neurotrophic receptor tyrosine kinase) fusions and *AXL*-related pathways. **Methods:** A total of 48 patients with NSCLC was analyzed, including 22 women and 26 men (mean age 70 years, range 44–86). Tumor specimens were classified histologically as adenocarcinomas (*n* = 81%) or squamous cell carcinomas (*n* = 19%). Smoking history, PD-L1 (programmed death-ligand 1) expression, and genetic alterations were assessed. NGS (Next-generation sequencing) identified genomic variants, which were classified according to ACMG (American College of Medical Genetics and Genomics) guidelines. **Results:** The cohort consisted of 29 former smokers, 13 current smokers, and 5 non-smokers (12%), with a mean smoking burden of 33 pack years. PD-L1 TPS (tumor proportion score) was ≥50% in 10 patients, ≥1–<50% in 22, and <1% in 15 patients. In total, 120 genomic variants were detected (allele frequency ≥ 5%). Of these, 52 (43%) were classified as likely pathogenic or pathogenic, 48 (40%) as variants of unknown significance, and 20 (17%) as benign or likely benign. The most frequently altered genes were *TP53* (tumor protein p53) (31%), *KRAS* and *EGFR* (15% each), and *STK11* (serine/threonine kinase 11) (12%). Adenocarcinomas accounted for 89% of all alterations, with *TP53* (21%) and *KRAS* (15%) being most common, while squamous cell carcinomas predominantly harbored *TP53* (38%) and *MET* (15%) mutations. In patients with PD-L1 TPS ≥ 50%, *KRAS* mutations were enriched (50%), particularly *KRAS* G12C and G12D, with frequent co-occurrence of *TP53* mutations (20%). No pathogenic EGFR mutations were detected in this subgroup. **Conclusions:** Comprehensive genomic profiling in NSCLC revealed a high prevalence of clinically relevant mutations, with *TP53*, *KRAS* and *EGFR* as the dominant drivers. The strong association of *KRAS* mutations with high PD-L1 expression, irrespective of smoking history, highlights the interplay between genetic and immunological pathways in NSCLC. These findings support the routine implementation of broad molecular testing to guide precision oncology approaches in both adenocarcinoma and squamous cell carcinoma patients.

## Simple Summary

Molecular diagnostics are central to NSCLC (Non-Small Cell Lung Cancer) management. Both the S3 guideline and the NCCN (National Comprehensive Cancer Network) recommend comprehensive NGS (Next-Generation Sequencing)-based profiling for all stage IV patients before therapy decisions. In addition to established biomarkers such as *EGFR* (epidermal growth factor receptor), *ALK* (anaplastic lymphoma kinase), *KRAS* (Kirsten rat sarcoma virus oncogene homologue), *BR*AF (B-Raf proto-oncogene serine/threonine kinase), *MET* (mesenchymal–epithelial transition factor), *RET* (rearranged during transfection), *ROS1* (ROS proto-oncogene 1), *NTRK* (neurotrophic receptor tyrosine kinase), and *HER2* (human epidermal growth factor receptor 2), emerging alterations such as *FGFR* (fibroblast growth factor receptor), *NRG1* (neuregulin 1), and *MET* exon 14 skipping or amplification should be assessed. PD-L1 (programmed death-ligand 1) testing is mandatory to guide immunotherapy decisions. Our cohort of 48 samples confirms the relevance of these biomarkers: *KRAS* mutations were most common (27%, with G12C the largest subgroup), while *EGFR* mutations occurred in 17% of cases, predominantly in never-smokers and women. *ALK* and *ROS1* fusions as well as *NTRK* alterations were not observed; rare occurrences included one *BRAF V600E*, one *MET* exon 14 mutation, and one *RET* mutation. *TP53* (tumor protein p53) mutations were frequent (~52%), often as a co-driver without targeted therapy options. Patient-related factors such as smoking status, sex, and PD-L1 expression strongly influenced biomarker patterns and treatment considerations: never-smokers were enriched for *EGFR* and *MET* alterations, whereas smokers showed higher prevalence of *KRAS*; women exhibited higher rates of *EGFR* mutations and higher PD-L1 expression, which may contribute to sex-specific differences in immunotherapy response. *STK11* [serine/threonine kinase 11] mutations clustered in PD-L1–negative tumors, supporting an immunosuppressive phenotype. Overall, the data align with guideline recommendations and underscore the importance of broad molecular profiling in NSCLC. Integrating genetic alterations with clinical features such as smoking history, sex, and PD-L1 status enables more precise patient stratification and personalized therapy.

## 1. Introduction

### 1.1. Lung Cancer as a Global Health Problem

Lung cancer remains a major global health burden and is the leading cause of cancer-related mortality worldwide. Among lung malignancies, non-small cell lung cancer (NSCLC) accounts for approximately 80–85% of cases and continues to represent a critical challenge despite advances in early detection and treatment. NSCLC is the leading cause of cancer-related death in men and the second leading cause in women globally [1,2]. Although short-term outcomes such as 12-month progression-free survival may be comparable, long-term survival remains poor, particularly among smokers, who exhibit nearly twice the risk of death within 5–10 years following diagnosis compared with non-smokers [3].

### 1.2. Global and Local Epidemiology and Mortality

Epidemiological data highlight the substantial burden of NSCLC across populations. According to the Surveillance, Epidemiology, and End Results (SEER) Program, the median age at lung cancer diagnosis in the United States is 71 years, reflecting the disease’s predominance in older adults [4]. Mortality rates remain high due to late-stage diagnosis, biological aggressiveness, and therapeutic resistance. While smoking-associated lung cancer incidence has declined in some regions, rising rates among women and never-smokers underscore the importance of environmental, occupational, and genetic risk factors.

### 1.3. Risk Factors

Tobacco smoking is the primary risk factor for lung cancer, accounting for approximately 85–90% of cases. However, a significant proportion of NSCLC arises in individuals without a smoking history. Additional risk factors include exposure to second-hand smoke, radon gas, asbestos, arsenic, chromium, beryllium, nickel, and other occupational carcinogens. Genetic susceptibility, positive family history, pulmonary fibrosis, HIV infection, and excessive alcohol consumption have also been linked to increased lung cancer risk [5]. These diverse etiological factors contribute to the molecular heterogeneity characteristic of NSCLC.

### 1.4. Types and Histological Classification

The classification of lung tumors follows the 2015 World Health Organization (WHO) guidelines [6]. NSCLC encompasses several histological subtypes, primarily adenocarcinoma, squamous cell carcinoma, and large cell carcinoma. Adenocarcinoma is the most prevalent subtype, accounting for approximately half of all lung cancer cases [1], particularly among never-smokers and women. Its diagnosis is based on neoplastic gland formation, expression of pneumocyte markers such as thyroid transcription factor-1 (TTF-1), with or without napsin A, and/or detection of intracytoplasmic mucin. Growth patterns include acinar, papillary, micropapillary, lepidic, and solid architectures.

In contrast, squamous cell carcinoma is characterized by keratin production and intercellular desmosomes, with immunohistochemical positivity for markers such as p40, p63, cytokeratin 5 (CK5), and desmoglein [7].

### 1.5. Molecular Alterations in NSCLC Pathogenesis

NSCLC development is driven by cumulative genetic and epigenetic alterations that disrupt normal cellular processes, including cell-cycle regulation, apoptosis, DNA repair, immune surveillance, and signaling pathway control. Carcinogen-induced DNA damage, defective repair mechanisms, and clonal selection result in malignant transformation and tumor progression. These processes underpin hallmark features of cancer, such as sustained proliferation, resistance to cell death, angiogenesis, invasion, metastasis, and immune evasion.

Multiple oncogenic drivers and tumor suppressor genes play central roles in NSCLC biology, Table 1. TP53, one of the most frequently mutated genes in NSCLC, regulates genomic integrity, apoptosis, and cell-cycle arrest; its inactivation promotes genomic instability and tumor aggressiveness. EGFR activating mutations lead to constitutive signaling through the MAPK and PI3K–AKT pathways, driving tumor growth and survival, particularly in adenocarcinoma. *KRAS* mutations, including the clinically actionable p.G12C variant, result in persistent downstream signaling and are associated with therapeutic resistance.

Gene rearrangements involving *ALK*, *ROS1*, *RET*, and *NTRK*, as well as alterations in *BRAF*, *MET*, and *ERBB2* (HER2), generate oncogenic fusion proteins or aberrant signaling pathways that sustain malignant phenotypes.

Immune evasion is mediated in part by PD-L1 (programmed death-ligand 1) expression on tumor cells, which suppresses antitumor T-cell activity via the PD-1 receptor, forming the biological basis for immune checkpoint inhibition (see Table 1).

### 1.6. Therapeutic Modalities and Precision Oncology

The identification of predictive and prognostic biomarkers has transformed NSCLC management. Molecular biomarkers such as *EGFR* mutations, *ALK* and *ROS1* fusions, *KRAS* p.G12C mutations, *MET* exon 14 skipping, and *BRAF* p.V600 alterations guide targeted therapy selection. PD-L1 expression and tumor mutational burden (TMB) inform immunotherapy eligibility and response prediction. Emerging biomarkers, including circulating tumor DNA (ctDNA), RNA-based fusion detection, and liquid biopsy approaches, offer non-invasive strategies for disease monitoring, resistance detection, and treatment adaptation.

Treatment options for NSCLC include surgery, radiotherapy, chemotherapy, immunotherapy, and molecularly targeted therapies. Over the past decade, molecular pathology has shifted NSCLC from a histology-driven disease toward a biomarker-driven precision oncology paradigm. Targeted therapies directed against *EGFR*, *ALK*, *ROS1*, *BRAF*, *KRAS G12C*, *MET*, *RET*, *NTRK*, and *NRG1* alterations have significantly improved outcomes in selected patient populations. Immune checkpoint inhibitors targeting the PD-1/PD-L1 axis have revolutionized the management of advanced NSCLC, particularly in tumors with high PD-L1 expression or elevated TMB.

In Germany, the National Network Genomic Medicine Lung Cancer (nNGM) ensures standardized, high-quality molecular diagnostics through immunohistochemistry (e.g., PD-L1) and comprehensive DNA- and RNA-based analyses, including next-generation sequencing (NGS), FISH, and fusion testing, in accordance with national guidelines [8,9,10].

**Table 1:** Summary of guideline-based biomarker–therapy associations [11,12,13,14,15,16,17,18].

Biomaker	Associated Therapy	Patient Population/Indication
*EGFR* (Exon 19 deletions, Exon 21 L858R, etc.)	EGFR Tyrosine Kinase Inhibitors (TKIs)	Advanced NSCLC, especially in non-squamous histologies, for patients with these specific mutations
*ALK* Fusions	ALK TKIs	Advanced NSCLC with *ALK* fusions
*ROS1* Fusions	ROS1 TKIs	Advanced NSCLC with *ROS*1 fusions
*BRAF p.V600* Mutations	BRAF/MEK Inhibitors	Advanced NSCLC with *BRAF V600* mutations
*KRAS p.G12C* Mutations	KRAS G12C Inhibitors	Advanced NSCLC with *KRAS G12C* mutations
*MET* Exon 14 Skipping Mutations	MET Inhibitors	Advanced NSCLC with *MET exon 14* skipping
*RET* Fusions	RET Inhibitors	Advanced NSCLC with *RET* fusions
*NTRK* Fusions	NTRK Inhibitors	Advanced NSCLC with *NTRK* fusions
*NRG1*	HER3 (ERBB3)-targeted therapies such as zenocutuzumab (MCLA-128)	Advanced NSCLC with *NRG1* fusions
PD-L1 Expression	Immune Checkpoint Inhibitors (ICIs)	Advanced NSCLC, particularly with high PD-L1 expression (e.g., TPS ≥ 50%)

### 1.7. Study Rationale

Despite these advances, challenges persist. Tumor heterogeneity, dynamic clonal evolution under treatment pressure, and variability in access to comprehensive molecular testing can impact clinical decision-making. Standardization of specimen handling, validation of robust molecular assays, and harmonization of reporting are essential to ensure equitable, high-quality care across diverse healthcare settings. This publication assessed the current state of NSCLC molecular pathology, outlines practical diagnostic and therapeutic workflows, and discusses future directions—including liquid biopsy, multi-omics integration, and real-world data—that hold promise for further personalizing management and improving survival for patients with NSCLC. The integration of comprehensive molecular profiling—via NGS, liquid biopsy, and multiplex assays—facilitates accurate histologic subclassification, prognostication, and selection of targeted therapies and immunotherapies. AI-based supportive software for molecular genetic variant interpretation supports clinicians and researchers by automating the classification of genetic alterations, integrating data from databases, the literature, and clinical evidence.

### 1.8. Study Aims

This study aims at:

Assessing the current state of molecular pathology in NSCLC within routine clinical practice.

Evaluate key molecular and immunological biomarkers relevant to diagnosis, prognosis, and therapeutic decision-making.

Outline practical diagnostic and therapeutic workflows and discuss future directions, including liquid biopsy, multi-omics integration, and AI-supported variant interpretation, to further personalize NSCLC management and improve survival.

## 2. Materials and Methods

### 2.1. Collective of Patients/Inclusion and Exclusion Criteria:

Formalin-fixed paraffin-embedded (FFPE) NSCLC samples from 26 men and 22 women aged 44 to 86 years (mean: 70 years, median: 71 years) were randomly included at the University Hospital Frankfurt/Main. The FFPE samples were collected from 2020 to 2021. Histologically, the samples were classified into nine squamous cell carcinomas (19%) and 39 adenocarcinomas (81%). The smoking status as well as the expression pattern of PD-L1 and variants of the core genes were assessed.

Inclusion criteria:

- Minimum tumor content of 15%.
- Histology: adenocarcinoma or squamous cell carcinoma

Exclusion criteria:

- Samples with tumor content below the threshold were excluded (*n*= 10)
- Insufficient sequencing parameters (*n* = 8):
  • % Q30 bases: <95%
  • Total number of reads: <50,000,000
  • Aligned reads: <95%
  • Coverage 500x: <95%
- Other histological subtypes (i.e., neuroendocrine tumors) were excluded (*n* = 6)

### 2.2. Molecular Diagnostics:

The DNA was extracted from FFPE tissue blocks after macrodissection using the QIAamp^®^ DNA Micro Kit (50) (Qiagen N.V., Venlo, The Netherlands) [19], the RNA with the help of the Maxwell^®^ RSC RNA FFPE Kit (Promega, Madison, WI, USA) [20] according to the manufacturer’s instructions, respectively. DNA quantification was performed using the 1X dsDNA Assay, for RNA quantification the RNA HS Assay was used [21], both on the Qubit™ fluorometer (ThermoFisher Scientific) [22].

For library preparation, 20 ng of DNA per sample, as specified for the panel, was used with the Oncomine Comprehensive Assay v3 (Thermo Fisher Scientific, Waltham, MA, USA). Library preparation was carried out in accordance with the manufacturer’s instructions. Clonal amplification and chip loading were performed using the Ion Chef™ System (Thermo Fisher Scientific). Sequencing was subsequently conducted on the Ion GeneStudio™ S5 System (Thermo Fisher Scientific). Sequencing data were analyzed using manufacturer-provided software platforms, with primary data processing performed using Ion Reporter™ software (Version 5.14).

Afterwards data were analyzed with the Ion Reporter™ software (version 5.12.0.0); filter chains Oncomine Variants 5.12 and Oncomine Extended 5.12 were used. The mutation status of following genes was evaluated:

*ALK*, *BRAF*, *CTNNB1*, *CUL3*, *EGFR*, *ERBB2 (*HER2*)*, *FGFR1*, *FGFR2*, *FGFR3*, *FGFR4*, *HRAS*, *IDH1*, *IDH2*, *KEAP1*, *KRAS*, *MAP2K1*, *MET*, *NFE2L2*, *NRAS*, *NTRK1*, *NTRK2*, *NTRK3*, *PIK3CA*, *PTEN*, *RB1*, *RET*, *ROS1*, *SMARCA4*, *STK11*, *TP53.*

Genomic alterations were identified by the alignment on the reference genome hg19 (GRCh37). To achieve reliable results, only alterations with fulfilled quality criteria were considered, such as allele frequency (AF) ≥ 5% and a coverage of at least 200× for the Ion S5™. Classification and interpretation of detected filtered and unfiltered variants were evaluated with MH Guide (v5.3, Molecular Health, Heidelberg, Germany), a CE-marked (IVDR 2017/746) tertiary NGS analysis software (Version 5.0). MH Guide identifies reportable variants and provides clinical interpretation, including potentially effective, ineffective, or high-risk medications. It offers variant annotation, classification, and interpretation based on a curated, peer-reviewed evidence database. This variant annotation provided by Molecular Health was manually reviewed according to the online databases ClinVar [23] and Cosmic [24]. Other databases used for variant interpretation were, i.e., gnomAD [25], OncoKB [26], dbSNP [27] and cBioPortal [28] (available online). For this study, the annotation of pathogenicity of the detected variants was determined according to the American College of Medical Genetics and Genomics (ACMG) classification in: “benign” (class 1), “likely benign” (class 2), “uncertain significance” (class 3), “likely pathogenic” (class 4), “pathogenic” (class 5). Classification is based on multiple evidence types: population frequency, computational predictions, functional assays, segregation data, de novo status, and published literature.

To achieve a consistent approach of naming all variants, sequence variant nomenclature was carried out in concordance with the guidelines by the Human Genome Variation Society (HGVS) [29].

Due to the small sample size per possible subgroup, statistical analysis should be interpreted with caution. Thus the following findings are descriptive and hypothesis-generating.

### 2.3. PDL1 Immunhistochemistry

PD-L1 expression was evaluated using the PD-L1 IHC 22C3 pharmDx kit (Agilent, Santa Clara, CA, USA, [30]) according to the manufacturer’s instructions. Typically the Tumor Proportion Score (TPS) was applied, which reflects the percentage of viable tumor cells showing partial or complete membrane staining at any intensity. Used thresholds:

TPS < 1% → PD-L1 negative
TPS 1–49% → PD-L1 low expression
TPS ≥ 50% → PD-L1 high expression

These thresholds are clinically relevant for treatment decisions (e.g., pembrolizumab eligibility in first-line NSCLC depends on TPS ≥ 50%).

### 2.4. Statistics

Statistics were obtained using IBM SPSS Statistics (Version 31). Statistical significance was defined as *p* < 0.05. Due to the small sample size per possible subgroup, statistical analysis should be interpreted with caution.

### 2.5. Ethics

## 3. Results

### 3.1. Cohort

**Figure 1:**
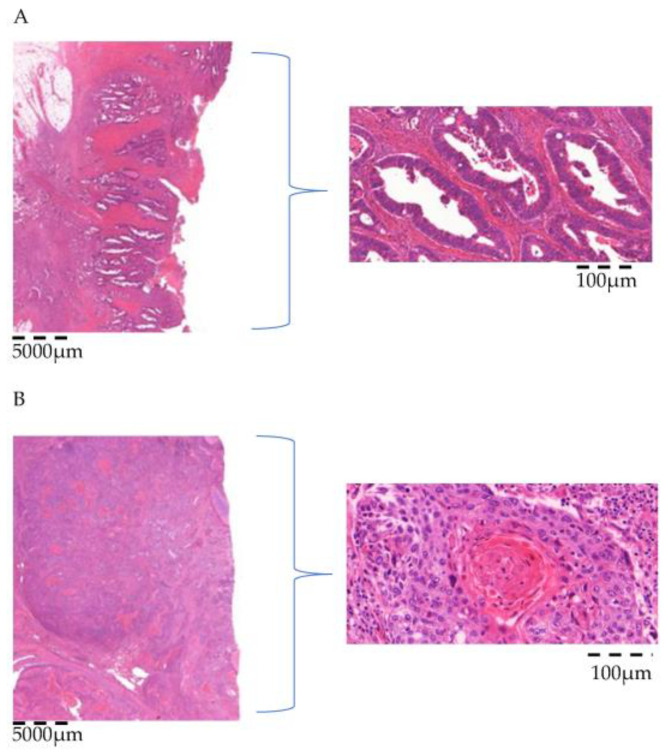
**Hematoxylin and eosin staining.** (**A**) Adenocarcinoma consisting of gland-forming epithelial cells and cytoplasmic mucin. (**B**) Squamous cell carcinoma usually presenting intercellular bridges, keratinization and a squamous (flat) cell morphology.

48 clinically diagnosed non-small cell lung cancers of 22 women (46%) and 26 men (54%) at the age of 44 to 86 years (mean: 70 years, median: 71 years) were analyzed. Histologically, nine pulmonary squamous cell carcinomas (19%) and 39 adenocarcinomas (81%) were assessed, (see Figure 1).

### 3.2. Smoking Status

**Figure 2:**
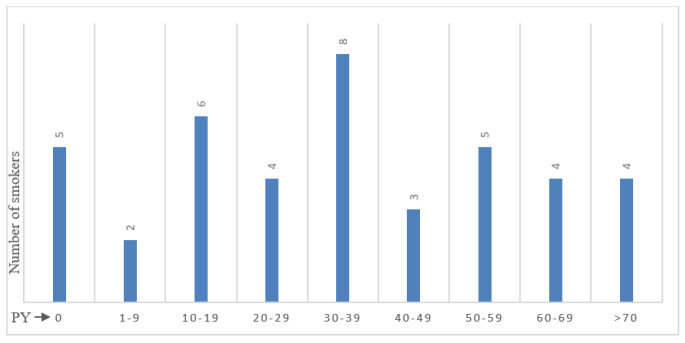
**Distribution of pack years (PY) within the patient cohort**. Two patients had 1–9 PY (5%), six patients 10–19 PY (15%), four patients 20–29 PY (10%), eight patients 30–39 PY (20%), three patients 40–49 PY (7%), five patients 50–59 PY (12%), four patients 60–69 PY (10%) and four patients > 70 PY (10%).

The smoking status of the patients was divided into 29 former smokers, 13, smokers and five non-smokers. No data was available for one patient. In the patient cohort, five patients (12%) were non-smokers (0 pack years (PY)). As visible in Figure 2 the remaining patients were distributed as follows: two patients 1–9 PY (5%), six patients 10–19 PY (15%), four patients 20–29 PY (10%), eight patients 30–39 PY (20%), three patients 40–49 PY (7%), five patients 50–59 PY (12%), four patients 60–69 PY (10%), four patients > 70 PY (10%). The pack-years average for the analyzed collective was 33.

### 3.3. PDL1-Status

**Figure 3:**
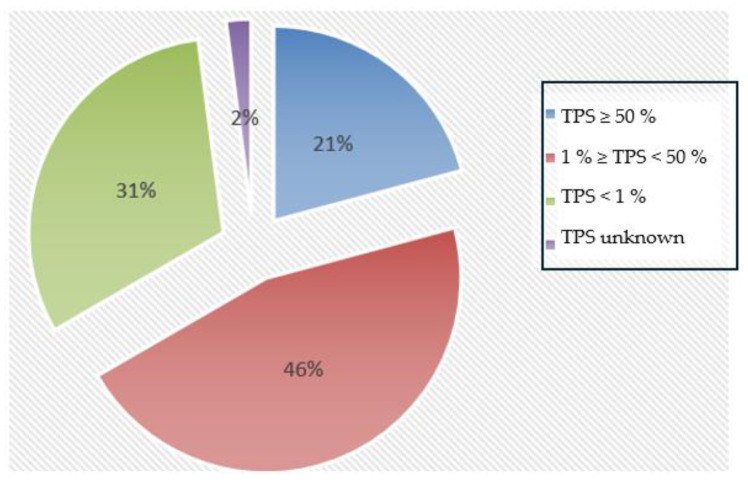
**Distribution of the assessed TPS thresholds within the patient cohort**. Ten patients showed a TPS of ≥50% (10/48, 21%), 22 patients had a TPS between ≥1% and <50% (22/48, 46%), 15 patients had a TPS of <1% (15/48, 31%). The score was unknown for one patient (1/58, 2%).

In the present studied cohort, a TPS score of ≥50% was found in ten patients (10/48, 21%), between ≥1% and <50% in 22 patients (22/48, 46%), and <1% in 15 patients (15/48, 31%). For one person (1/48, 2%), the TPS score was unknown (see Figure 3).

### 3.4. Distribution of Genetic Alterations (ACMG Classes 1–5)

**Figure 4:**
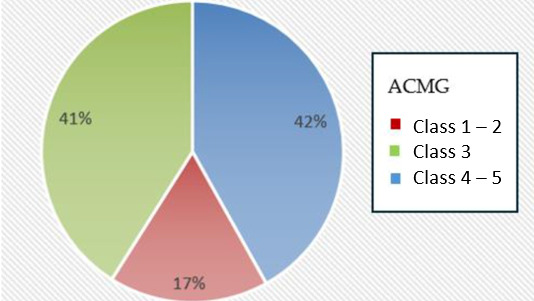
**Distribution of the genetic alterations identified in the patient population**. Assessment of their pathogenicity was conducted according to ACMG criteria, including only variants with allele frequency ≥ 5%. 120 alterations were detected in total. 42% (52/120) were categorized as likely or clearly pathogenic (ACMG classes 4 and 5), 41% (48/120) were determined to be variants of unknown significance (ACMG class 3) and 17% (20/120) were classified as benign or likely benign (ACMG classes 1 and 2).

The identified genetic alterations (*n* = 120, Figure 4), were annotated with regard to their potential pathogenicity and clinical relevance using the ClinVar database (National Library of Medicine, Bethesda, MD, USA). Therefore, 52 variants (52/120, 42%) were categorized as likely (LP, ACMG class 4) or clearly pathogenic (P, ACMG class 5) mutations, 48 variants (48/120, 41%) were determined to be variants of unknown significance (VUS, ACMG class 3) and 20 variants (20/120, 17%) were classified as benign (B) or likely benign (LB) (ACMG classes 1 and 2).

**Figure 5:**
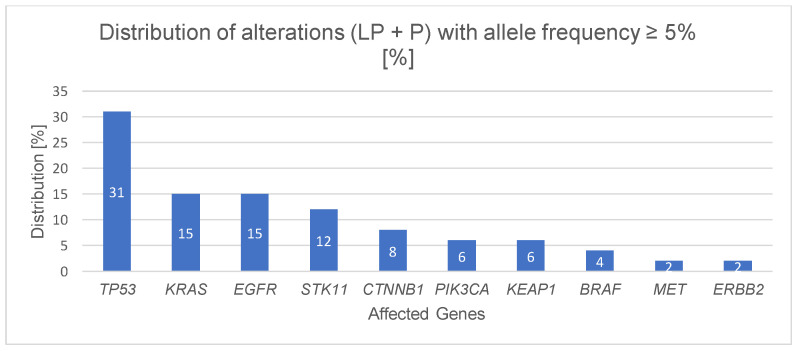
**Distribution of likely pathogenic (LP)/pathogenic (P) alterations ≥ 5% AF in the patient cohort**. The most affected gene was *TP53* (31%, 16/52), followed by *KRAS* and *EGFR* (both 15%, 8/52), *STK11* (12%, 6/52), *CTNNB1* (8%, 4/52), *PIK3CA*, *KEAP1* (both 6%, 3/52), *BRAF* (4%, 2/52) and *MET* and *ERBB2* (both 2%, 1/52).

52 alterations (see Figure 5), assigned as likely pathogenic (LP) and pathogenic (P) for ADC and SQC with a allele frequency of ≥5% are distributed across 10 genes: *TP53* (31%, 16/52), *KRAS* and *EGFR* (both 15%, 8/52), *STK11* (12%, 6/52), *CTNNB1* (8%, 4/52), *PIK3CA*, *KEAP1* (both 6%, 3/52), *BRAF* (4%, 2/52), *MET*, *ERBB2* (both 2%, 1/52).

Broken down by histological tumor entity, the 39 adenocarcinomas (ADC) accounted for 107 of the total of 120 alterations (89%). 13 were detected in the squamous cell carcinomas (SQC). *TP53* was affected most in both subgroups: 21% (22/107) of ADCs and 38% (5/13) in SQCs showed a mutation there. The second most common affected gene was *KRAS* in ADCs and *MET* in SQCs (15% each). The rest of the variant distribution can be found in Table 2.

**Figure 6:**
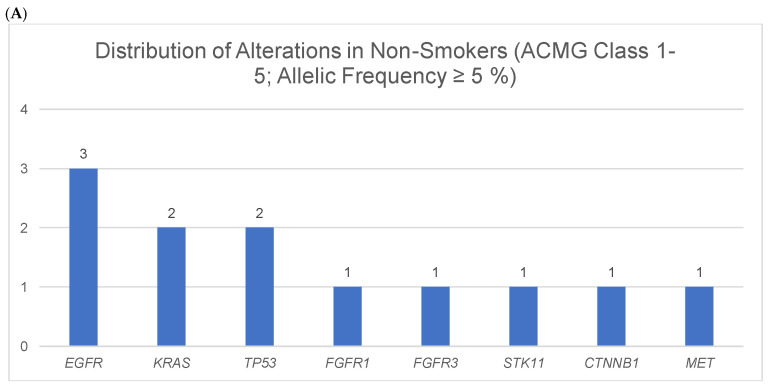

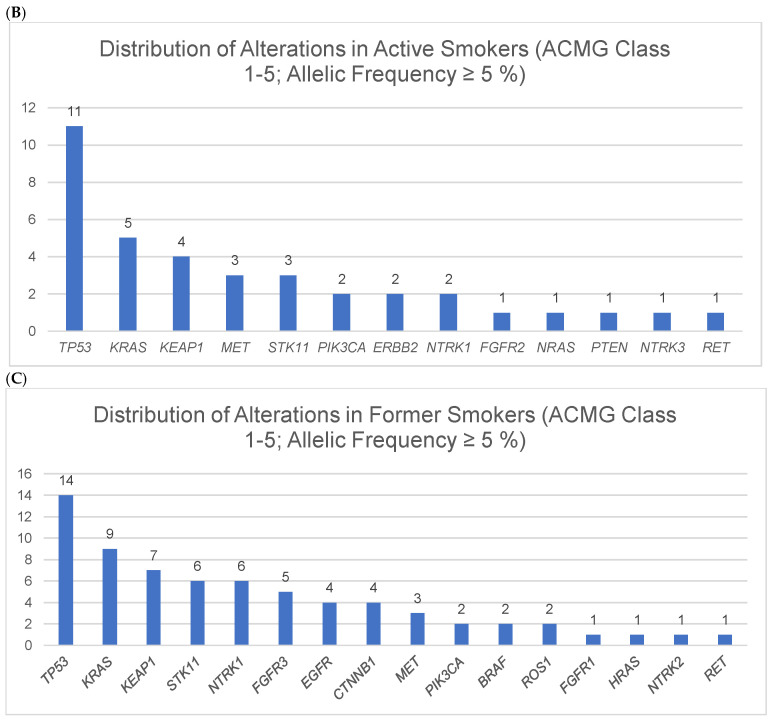
**(A–C): Distribution of alterations in non-smokers, active and former smokers. **(**A**) Non-smokers (classes 1–5 according to ACMG criteria) with allele frequency of ≥5%. In non-smokers, a total of 12 alterations was identified across eight genes: *EGFR* (*n* = 3), *KRAS* and *TP53* (*n* = 2), and *FGFR1*, *FGFR3*, *STK11*, *CTNNB1*, and *MET* (*n* = 1). (**B**) Active smokers (classes 1–5 according to ACMG criteria) with allele frequency of ≥5%. In active smokers, the analysis revealed a total of 37 alterations distributed across 13 genes: *TP53* (*n* = 11), *KRAS* (*n* = 5), *KEAP1* (*n* = 4), *STK1*1 and *MET* (*n* = 3 each), *PIK3CA*, *ERBB2*, and *NTRK1* (*n* = 2 each), as well as *FGFR2*, *NRAS*, *PTEN*, *NTRK3*, and *RET* (*n* = 1 each). (**C**) Former smokers (classes 1–5 according to ACMG criteria) with allele frequency of ≥5%. In former smokers, 68 alterations were detected across 16 genes: *TP53* (*n* = 14), *KRAS* (*n* = 9), *KEAP1* (*n* = 7), *STK11*, *NTRK1* (both *n* = 6), *FGFR3* (*n* = 5), *EGFR*, *CTNNB1* (both *n* = 4), *MET* (*n* = 3), *PIK3CA*, *BRAF*, *ROS1* (all *n* = 2), *FGFR1*, *HRAS*, *NTRK2*, *RET* (all *n* = 1).

In the cohort, there were five patients who had never smoked in their lifetime. In the analysis of these non-smokers, 12 alterations were identified (2.4 alterations per patient (12/5)), Figure 6A). This corresponds approximately to the mutation burden of the remaining cohort (2.5 alterations per patient).

The group of active smokers comprised 13 patients who exhibited a total of 37 alterations. This corresponded to a mutation burden of 2.8 alterations per patient, representing an increase compared to the rest of the cohort (2.4 alterations per patient). The exact distribution is shown in Figure 6B.

For non-smokers the alterations are classified into nine pathogenic variants (*EGFR* (3), *KRAS* (2), *TP53* (2), *STK11* (1), and *CTNNB1* (1)), one variant of uncertain significance (VUS) (*FGFR1* (1)) and benign ones (*MET* (1) und *FGFR3* (1)).

The subcohort of former smokers comprised 29 patients with a total of 69 detected alterations. This corresponded to a mutation burden of 2.4 alterations per patient, which was identical to that of the non-smoker group (see Figure 6C).

For smokers, 13 benign alterations were identified (*FGFR3* (6), *NTRK1* (4), *MET* (2), *KEAP1* (1)). 35 variants were classifies as pathogenic and 20 as VUS, (see Figure 6B). Notably, an increased occurrence of *EGFR* alterations was observed among non-smokers compared to smokers: three out of five non-smoking patients exhibited *EGFR* mutations.

There are considerable differences between non-smokers and current or former smokers in terms of treatment options. Four out of five non-smokers could benefit from a personalized therapy (80%), whereas only nine out of 43 patients in the remaining cohort (21%) could do so. At the mutation level, four out of nine pathogenic alterations in non-smokers were actionable (44%), compared to nine out of 61 pathogenic alterations in smokers and former smokers (15%), denoting a statistically significant difference between ever- and never-smokers (*p* < 0.05). Due to the small sample size per possible subgroup, statistical analysis should be interpreted with caution.

## 4. Discussion

In our study 52 genetic alterations were classified as likely pathogenic (LP) or pathogenic (P) in adenocarcinoma (ADC) and squamous cell carcinoma (SQC) with an allele frequency of ≥5%. These alterations were distributed across ten genes: *TP53* (31%; 16/52), *KRAS* and *EGFR* (each 15%; 8/52), *STK11* (12%; 6/52), *CTNNB1* (8%; 4/52), *PIK3CA* and *KEAP1* (each 6%; 3/52), *BRAF* (4%; 2/52), and *MET* as well as *ERBB2* (each 2%; 1/52). In contrast to *KRAS*, *EGFR*, *BRAF*, *MET* and *ERBB2* no approved targeted therapy is yet available for *STK11*, *CTNNB1*, *PIK3CA* and *KEAP1.* These genes have growing relevance for prognosis and treatment stratification in NSCLC. *STK11* and *KEAP1* mutations, mainly in adenocarcinoma, are linked to poor outcomes and reduced immunotherapy response. *CTNNB1* mutations activate the Wnt/β-catenin pathway and contribute to immune resistance. *PIK3CA* alterations occur in both adenocarcinoma and squamous carcinoma, activating the PI3K/AKT/mTOR pathway and representing potential targets for PI3K inhibitors [31,32,33,34,34,35,36,37,38,39,40]. *TP53*, which is involved in numerous biological processes and associated with tumor progression, metastasis, reduced chemo- and radiotherapy response as well as decreased patient survival, is among the most frequently mutated genes in NSCLC, with mutation frequencies of 34–46% in adenocarcinomas and 65–81% in squamous cell carcinomas.

Alcohol and environmental exposures, including radon, asbestos, and air pollution, further contribute to malignant transformation through DNA damage, oxidative stress, and chronic inflammation [3]. Tobacco smoking is the primary driver of lung carcinogenesis, inducing DNA damage, mutations in key oncogenes and tumor suppressors, and a tumor-promoting microenvironment [1,2].

Thus smoking is a known risk factor, with risk increasing alongside pack years. Therefore, smoking status was also evaluated in correlation with mutation status. In the present cohort we found slightly higher *TP53* mutation prevalence in never-smokers than in smokers with <20 pack years (never-smokers 40% vs. <20 pack years 37.5%). This supports the hypothesis that smoking-induced *TP53* mutations represent a biologically distinct tumor entity compared with *TP53* mutations in never-smokers. One study reported higher *TP53* mutation prevalence in never-smokers than in smokers with <20 pack years (36% vs. 31%), which correlated with poorer survival in never-smokers [41]. At present, no targeted therapy exists for *TP53*-mutant NSCLC.

The present study shows a *KRAS* mutation prevalence of 38% (15/39) in adenocarcinomas. No *KRAS* mutations were found in squamous cell carcinomas. In accordance with our data, *KRAS* which upregulates proliferation, translation, and cell survival, is also one of the most frequently altered genes, with a prevalence of 15–33% in Western adenocarcinoma cases. In squamous cell carcinomas, *KRAS* mutations are less common (3% of cases). The most frequent subgroup of all *KRAS* alterations is the point mutation G12C, which accounts for 39% of all *KRAS* alterations [42]. This mutation was detected in the presente cohort in both smokers and non-smokers. The fact that two non-smokers also exhibited *KRAS* mutations indicates that, in addition to exogenous factors such as tobacco use, endogenous mechanisms, such as differences in tumor biology, may also play a role in the development of *KRAS* mutations. Data on the prognosis of NSCLC cases harboring *KRAS* mutations are contradictory, with studies suggesting both better and worse outcomes. Currently, five *KRAS* inhibitors are in clinical trials, three of which have been developed specifically for G12C mutations [43]. Guidelines recommend a targeted therapy with the currently only approved drug, Sotorasib, for *KRAS* G12C alterations as second-line treatment after failure of chemo-immunotherapy. Response rates of 36% and median duration of response of 10 months have been reported [44]. Six *KRAS* p.G12C cases were detected in the cohort which could pententially benefit from targeted therapy with Sotorasib. This could not be confirmed in the investigated cohort (*KRAS* patients: mean 69.1 years vs. overall cohort mean 69.9 years). The small cohort size may explain this distribution, as statistically significant associations are harder to prove and random variation has a greater impact. The link between *KRAS* mutation frequency and smoking behavior is well established. The likelihood of *KRAS* alterations increases in former smokers and rises further in active smokers [45]. The probability peaks at 16 pack years and remains constant thereafter, regardless of higher exposure. These correlations could not be demonstrated in the present cohort. Although smokers were overrepresented in the *KRAS* mutation group (5/13; 38%), former smokers were underrepresented (9/29; 31%). Interestingly, never-smokers were proportionally the most affected (two of five2/5; 40%).

In this patient cohort, eight pathogenic alterations were identified (17% of cases) in the *EGFR* gene. Seven were found in adenocarcinomas (18% (7/39) of adenocarcinoma cases) and one in a squamous cell carcinoma (11% (1/9) of squamous cell carcinoma cases). *EGFR* mutations are much less frequent in squamous cell carcinomas.

The discrepancy in the observed incidence in this cohort compared to the literature may be explained by the limited sample size in the squamous cell carcinoma subgroup, which may bias the incidence rate.

Point mutations in exon 21 at codon 858 (L858R) and deletions in exon 19 belong to the sensitizing mutations, meaning that the alteration itself enables drug responsiveness to EGFR tyrosine kinase inhibitors (TKIs) improving progression-free survival and quality of life [46]. *EGFR* mutations are considerably more common in never-smokers (42%). The literature reflects this trend: the probability of *EGFR* alterations is highest in never-smokers and decreases in former and current smokers, with an inverse correlation between pack years and *EGFR* mutation probability [47]. Furthermore, higher mutation rates have been described in female patients [48]. In this cohort, *EGFR* mutations were more frequent in female patients as well (five vs. three).

First-line therapy for *EGFR*-positive tumors (L858R point mutation in exon 21) includes EGFR tyrosine kinase inhibitors as recommended in the guidelines [49]. For exon 19 deletions, osimertinib is preferred due to superior survival outcomes. For uncommon *EGFR* mutations, afatinib or osimertinib is recommended [50]. The L858R point mutation and exon 19 deletions account for 90% of *EGFR* mutations in NSCLCs, while the remaining uncommon mutations represent a heterogeneous group of alterations in exons 18–21 [51]. A frequent resistance mechanism following targeted therapy is the exon 20 T790M point mutation, detected in about 50% of patients resistant to first- and second-generation TKIs [52]. Osimertinib (a third-generation TKI) is recommended as second-line therapy or, if present at baseline, as first-line therapy [53]. Liquid biopsy allows minimally invasive detection of circulating tumor DNA, CTCs, and exosomal nucleic acids, capturing tumor heterogeneity and enabling real-time monitoring of treatment response and resistance [4,5]. It is particularly valuable when tissue biopsies are limited and supports rapid, clinically actionable molecular profiling.

In this cohort Icotinib was suggested for *EGFR* p.E746_A750del (4 cases), *EGFR* p.L858R, and *EGFR* p.L747_P753delinsS (1 case each). Compared to the TKIs recommended in German guidelines (e.g., gefitinib or erlotinib), icotinib appears to have advantages in terms of side effects; however, overall survival is not improved. For *EGFR* p.S768_D770dup (1 case). A combination of mobocertinib and poziotinib was recommended. In German guidelines, no targeted first-line therapy is currently recommended for *EGFR* exon 20 insertions, including *EGFR* p.S768_D770dup. As second-line therapy, treatment with the monoclonal antibody amivantamab is advised. Mobocertinib, approved in the US and China, has shown a favorable risk–benefit profile, with a response rate of 28% in a study of chemotherapy-pretreated *EGFR* exon 20–positive tumors [54]. Poziotinib has demonstrated antitumor activity in *HER2* exon 20 (*ERBB2*) insertions but is associated with significant side effects such as rash, diarrhea, and stomatitis [55]. For this reason, the FDA has not granted approval for poziotinib in the US.

In this cohort no RNA-based *ALK* or *ROS1* fusions were detected. According to the literature, an *ALK* rearrangement rate of 3–5% has been described in adenocarcinomas [56]. *ALK* fusions (*EML4-ALK*, or fusions partners such as *KIF5B* or *HIP1*) are extremely rare in squamous cell carcinomas and are often limited to case reportsMoreover, the frequency of *ALK* fusions decreases steadily with increasing patient age [57]. Nonsmokers are also significantly more likely to harbor *ALK* fusions [58]. This may point to different biological mechanisms between smokers and nonsmokers in the pathogenesis of NSCLCs. Current guidelines recommend treatment with preferred *ALK* inhibitors such as alectinib, brigatinib, or lorlatinib for ALK-positive patients. In the event of treatment failure with a first-generation ALK inhibitor (crizotinib), a second-generation ALK inhibitor (ceritinib, alectinib, brigatinib) should be used as second-line therapy. After failure of second-generation ALK inhibitors, lorlatinib, the first third-generation agent, can be administered [59,60]. Additionally to *ALK* fusions, *ROS1*, which encodes a receptor tyrosine kinase that becomes also constitutively active through chromosomal rearrangement and fusion with other genes, promotes cellular transformation. One study identified nine different *ROS1* fusion partners, with CD74 being the most frequent [61]. *ROS1* translocations rarely co-occur with other alterations (EGFR, KRAS, ALK), suggesting an independent oncogenic role. The exact mechanism of *ROS1* oncogenicity is unclear, but activation of PI3K and MAPK signaling is suspected, ultimately driving cell growth and survival [62]. *ROS1* translocations occur in 1–2% of NSCLC cases, but are rare in squamous cell carcinoma [63].

FISH and immunohistochemistry are gold standards for *ALK* and *ROS1* translocation detection, while NGS workflows represent a valid alternative with broader mutation coverage. Like *EGFR* mutations and *ALK* translocations, *ROS1* fusions are enriched in never-smokers. A higher prevalence has also been observed in younger patients, with incidences of 6% reported in cohorts under 40 years old [64]. Guidelines recommend Crizotinib or Entrectinib as first-line treatment. As second-line therapy, Lorlatinib (off-label) is suggested, except in the presence of a G2032R resistance mutation, in which case platinum-based chemotherapy or enrollment in a clinical trial of next-generation ROS1 inhibitors is recommended [65]. In this patient cohort, a pathogenic *BRAF* alteration was identified in adenocarcinomas corresponding to 2% of patient cases. No *BRAF* mutation was found in squamous cell carcinomas. The patient diagnosed with a *BRAF* alteration in this cohort was a former smoker with a below-average number of pack years (20 vs. a mean of 33.1 pack years). In the present cohort, the only V600E mutation was detected in a female patient.

This incidence falls within the range reported in the literature (1–10%). *BRAF* which plays an important role in proliferation and cell survival, is very rare alterated in squamous cell carcinomas (0.3%) [66]. Approximately 30–40% of *BRAF* mutations are point mutations in exon 15 (V600E), which lead to increased kinase activity [40,67]. This subtype is diagnosed more frequently in women than in men (8.6% vs. 0.9%) [68]. On a biological level, *BRAF* V600E–mutated NSCLCs exhibit a more aggressive histological phenotype, which is associated with poorer prognosis [66].

A meta-analysis investigating the association between smoking status and *BRAF* alterations found no significant difference in the overall frequency of *BRAF* mutations among current, former, and never smokers. However, when considering only *BRAF* V600E mutations, never smokers were significantly more frequently affected than smokers or former smokers [69]. Targeted therapy in this setting consists of a combination treatment with dabrafenib (a BRAF inhibitor) and trametinib (a MEK inhibitor), thereby inhibiting two proteins within this signaling pathway [70].

In the present cohort, one adenocarcinoma case was found to harbor a *MET exon 14* point mutation (2% of cases). The patient identified in this study had these demographic characteristics: an 80-year-old female non-smoker, significantly older than the mean age (69.9 years). Larger cohorts have shown that patients with *MET exon 14* mutations are typically older, more often female, and mutually exclusive of *KRAS* and *EGFR a*lterations [71]. In *METex14* alterations, the pathophysiological mechanism involves constitutive activation of the MET receptor due to impaired receptor internalization. This results in sustained *MET* signaling, leading to proliferation and tumor growth. *MET* amplifications arise from an increased number of chromosome 7 copies, which harbors the *MET* gene. The basis of *MET* overexpression is *MET* receptor overactivation. In adenocarcinomas, *MET* alterations are reported in 7% of cases, with prevalence distributed as 3–4% *METex14* alterations and 1–2% *MET* amplifications. Squamous cell carcinomas show similar frequencies [15]. Currently there seems to be no clear coherence in *MET* alteration prevalence linked to smoking status. Upon confirmation of *MET exon 14* skipping, therapy with Capmatinib, Tepotinib, or Crizotinib is recommended according to NCCN guidelines.

In the studied cohort, no pathogenic *NTRK* fusion was detected. The *NTRK* gene family (*NTRK1*, *NTRK2*, *NTRK3*) encodes tropomyosin receptor kinases (TRK), which function as receptors for neurotrophins (nerve growth factors). They activate, among others, the RAS cascade and PI3 kinase, thereby promoting cell proliferation and survival [72]. A meta-analysis across tumor entities found a prevalence of 0.2% for *NTRK* fusions in lung carcinomas. Fusions involving *NTRK2* and *NTRK3* occur in < 1% of lung adenocarcinomas. In one cohort without detectable driver mutations, *NTRK1* fusions were observed in 3.3% of cases, with *MPRIP* or *CD74* fusion partners, leading to constitutive activation of signaling pathways and oncogenic effects [73]. NCCN guidelines recommend treatment with Larotrectinib or Entrectinib in *NTRK1-3*-positive lung cancers.

In the current cohort, a pathogenic *RET* point mutation (2% of cases) was identified in an adenocarcinoma. The identified patient was younger with a little smoking history as compared to the average age and mean pack years of the cohort. (51 years vs. cohort mean 69.9 years, 30 pack years vs. mean 33.1). *RET* fusions are detected in 1–2% of NSCLC cases [74,75], but are very rare in squamous cell carcinoma. Literature suggests *RET* alterations are more common in younger patients with little or no smoking history [76]. The *RET* gene encodes a receptor tyrosine kinase at the beginning of the RAS-RAF-MEK-ERK pathway. Genetic fusions with partners such as *KIF5B*, *CCDC6*, or *TRIM33* act as driver mutations via aberrant, ligand-independent *RET* activation. The *KIF5B* fusion is the most common, resulting from an inversion on chromosome 1 *RET*-positive patients should be treated with RET inhibitors such as Selpercatinib or Pralsetinib (NCCN).

In the examined cohort of 48 patients PD-L1 status was evaluated according to the common accepted guideline [77,78]. Ten patients had a PDL1 TPS score of ≥50%.

The analysis of the correlation between PD-L1 expression and detected genetic alterations in this cohort revealed distinct patterns. 30% of patients with high PD-L1 expression showed pathogenic *TP53* mutations, and co-mutations in *KRAS* and *TP53* were detected in 20% of patients. An *FGFR1* mutation was found in one additional patient. In the PD-L1–negative group (TPS < 1%), *EGFR* mutations were detected in 14% of cases (2/14 patients), whereas in the PD-L1–positive group (TPS ≥ 1%), 18% of patients (6/33) harbored *EGFR* mutations. Interestingly, no pathogenic *EGFR* mutations were detected in any of the patients with a TPS ≥ 50%. Some studies postulate a potential correlation between *EGFR* mutations and high PD-L1 expression in specific subgroups of NSCLC patients. In contrast, other studies support the hypothesis of an inverse relationship between these two factors [79,80]. The prevalence of *KRAS* mutations was comparable between the two subgroups (PD-L1–negative: 36%, 5/14; PD-L1–positive: 33%, 11/33). Overall, 50% of patients with a PDL1 TPS score of ≥50% had a *KRAS* mutation, with the *KRAS-G12C* and *KRAS-G12D* variants (each representing two of the five KRAS mutations) being the most frequent. This subgroup had the highest incidence of *KRAS* mutations. The proportion of *KRAS G12C* mutations between PDL1 negative and positive carcinomas was also similar (PD-L1–negative: 40%, 2/5; PD-L1–positive: 36%, 4/11). Studies examining the association between *KRAS* mutations and PD-L1 status demonstrate heterogeneous results. Some study groups reported an increased frequency of *KRAS* mutations with high PD-L1 expression, while other cohorts failed to confirm this association [81,82]. These discrepancies suggest that the relationship between *KRAS* mutations and PD-L1 expression may be influenced by additional biological and molecular mechanisms that manifest differently across study populations.*TP53* mutations occurred slightly less frequently in the PD-L1–negative group compared to the PD-L1–positive group (57% vs. 61%). Previous studies have demonstrated that patients with higher PD-L1 expression more frequently harbor *TP53* mutations [83]. In contrast, a striking finding was the high mutation rate of *STK11* in PD-L1–negative patients (43% vs. 12%). Other studies have also reported a correlation in this context. Loss of *STK11* function has been linked to an immunosuppressive tumor phenotype, resulting in reduced PD-L1 expression on the cell surface [84]. Notably, certain uncommon molecular patterns, such as *STK11* enrichment in PD-L1–negative tumors, underscore the complexity of NSCLC biology and the need for comprehensive profiling beyond standard biomarkers. With respect to *KRAS/TP53* co-mutations, the PD-L1–positive group showed a slightly higher frequency compared to the PD-L1–negative group (15% vs. 14%). The correlation between this co-mutation and PD-L1 expression has been demonstrated in previous research. It is well established that *TP53* mutations influence a wide range of regulatory processes controlling cell survival. In combination with *KRAS* mutations, synergistic effects may arise that enhance tumor cell survival capacity and potentially increase PD-L1 expression. This, in turn, may promote immune evasion and thereby improve tumor survival. Immune checkpoint inhibitors targeting PD-1, PD-L1, and CTLA-4 restore antitumor immunity and can produce durable responses with a favorable toxicity profile [6,7]. Biomarkers such as PD-L1 and tumor mutational burden allow patient stratification, and combination therapies expand clinical applicability.

According to the literature smokers and former smokers tend to exhibit higher PD-L1 expression in tumor cells, which is associated with a better response to immunotherapies [85]. The likely explanation is a higher tumor mutational burden, which increases the probability of elevated PD-L1 expression—a positive predictor for the success of anti–PD-1 therapies. Moreover, it has been shown that immunotherapy with pembrolizumab (for TPS scores > 50%) demonstrates greater efficacy in former smokers compared to active smokers. In addition, evidence suggests that smoking cessation during therapy may prolong survival [86,87]. A meta-analysis investigating sex-specific differences in the efficacy of immunotherapies demonstrated that women tend to respond better to these treatments than men [88]. This may be explained, among other factors, by sex-specific differences in immune response, tumor biology, and pharmacological drug activity. Furthermore, it has been shown that PD-L1 expression is higher in women with NSCLC compared to men [89]. Another sex-specific difference is observed in the risk of developing lung cancer as a smoker. A Norwegian study, stratifying by pack-years in increments of 10, calculated a 43% increased risk for men and a 64% increased risk for women [90]. These results suggest that there are sex-specific biological differences in the response to tobacco-related carcinogens, making women more susceptible to the carcinogenic effects of smoking despite equal cigarette consumption. A possible explanation is the interaction between exogenous tobacco-derived toxins and female sex hormones, which may enhance carcinogenic potential in women.

As shown by own data smoking status, sex, and PD-L1 expression play also a decisive role for the assessment of therapy and prognosis. According to current German S3 and NCCN guidelines, all patients with stage IV NSCLC should undergo comprehensive NGS-based molecular diagnostics prior to first-line therapy [91,92], including broad genotyping in patients with wild-type *EGFR*, *ALK*, *ROS1*, *BRAF V600*, *NTRK*, *RET*, and *METex14*. This includes both well-established driver mutations (*EGFR*, *ALK*, *KRAS G12C*, *ROS1*, *BRAF V600E*, *NTRK1/2/3*, *MET exon 14 skipping*, *RET*, *HER2*, *NRG1*) and emerging biomarkers (high-level *MET* amplifications, *FGFR* alterations). In addition, PD-L1 testing is recommended [20] to guide therapy decisions and eligibility for clinical trials.

This study has several limitations that should be acknowledged. First, the cohort size was relatively small, and detailed clinical information for the included NSCLC patients was limited. Beyond basic demographic and histopathological data—such as age, sex, tumor localization, and diagnosis—comprehensive clinical follow-up information, including disease stage, treatment history, therapeutic response, and survival outcomes, was not available. This restricts the ability to correlate molecular or pathological findings with prognostic or treatment-related parameters. The lack of extended clinical data primarily reflects the retrospective, multi-institutional nature of sample collection, where full clinical documentation was not consistently accessible. Due to the small sample size per possible subgroup, statistical analysis should be interpreted with caution. Thus the findings are descriptive.

Second, matched-normal samples were not available, limiting the ability to definitively distinguish somatic variants from germline alterations. Technical constraints also exist, including potential artifacts associated with formalin-fixed, paraffin-embedded (FFPE) tissue as well as the possibility of batch effects or temporal heterogeneity could influence variant detection and allele frequencies. These factors should be carefully considered when interpreting variant prevalence, pathway involvement, and potential clinical implications.

## 5. Conclusions

Comprehensive molecular diagnostics, including NGS, PD-L1 assessment, and liquid biopsy, are essential for guiding personalized therapy in NSCLC. Automated workflows and molecular tumor boards enable precise identification of actionable biomarkers, including *EGFR*, *KRAS*, *ALK*, *BRAF*, *MET*, *RET*, *NTRK*, *ROS1*, and *TP53*, and emerging markers like *NRG1* and *FGFR*. Mutation prevalence varies with smoking status, sex, and age, while PD-L1 expression and co-mutations highlight the complexity of predicting therapeutic response. Timely molecular testing is critical for selecting targeted therapies such as *KRAS* G12C and RET inhibitors. Larger, well-annotated studies are needed to validate these approaches and further refine precision oncology for optimal patient outcomes.

## Figures and Tables

**Table 2:** Percentage distribution of alterations in ADCs and SQCs (ACMG classes 1–5) with allele frequency ≥ 5%.

Gene	Distribution of Alterations (Class 1–5)	
	ADC [%], *n* = 39	SQC [%], *n* = 9
*TP53* (*p* < 0.05)	21	38
*KRAS* (*p* < 0.05)	15	
*KEAP1* (*p <* 0.05)	10	
*STK11* (*p* > 0.05)	8	8
*EGFR* (*p* > 0.05)	7	8
*FGFR3* (*p* > 0.05)	7	8
*NTRK1* (*p* > 0.05)	7	8
*MET*(*p* > 0.05)	5	15
*CTNNB1* (*p* < 0.05)	5	
*PIK3CA* (*p* < 0.05)	3	8
*BRAF* (*p* < 0.05)	2	
*ROS1* (*p* < 0.05)	2	
*RET* (*p* < 0.05)	2	
*ERBB2* (*p* < 0.05)	2	
*PTEN* (*p* < 0.05)	1	
*FGFR2* (*p* < 0.05)	1	
*NRAS* (*p* < 0.05)	1	
*FGFR1* (*p* < 0.05)	1	
*HRAS* (*p* < 0.05)	1	
*NTRK2* (*p* < 0.05)	1	
*NTRK3* (*p* < 0.05)		8

## Data Availability

The original contributions presented in this study are included in the article. Further inquiries can be directed to the corresponding authors.
